# Reliability of diagnosis and clinical efficacy of visceral osteopathy: a systematic review

**DOI:** 10.1186/s12906-018-2098-8

**Published:** 2018-02-17

**Authors:** Albin Guillaud, Nelly Darbois, Richard Monvoisin, Nicolas Pinsault

**Affiliations:** 1 CORTECS team, University of Grenoble-Alpes, Paris, France; 2 ThEMAS team, TIMC-IMAG laboratory, UMR CNRS-UGA, 5525 Grenoble, France; 3 School of Physiotherapy, Grenoble-Alpes University Hospital, Paris, France; 4 Critical Thinking Research Federation, University of Grenoble-Alpes, FED, 4270 Paris, France

## Abstract

**Background:**

In 2010, the World Health Organization published benchmarks for training in osteopathy in which osteopathic visceral techniques are included. The purpose of this study was to identify and critically appraise the scientific literature concerning the reliability of diagnosis and the clinical efficacy of techniques used in visceral osteopathy.

**Methods:**

Databases MEDLINE, OSTMED.DR, the Cochrane Library, Osteopathic Research Web, Google Scholar, Journal of American Osteopathic Association (JAOA) website, International Journal of Osteopathic Medicine (IJOM) website, and the catalog of *Académie d’ostéopathie de France* website were searched through December 2017. Only inter-rater reliability studies including at least two raters or the intra-rater reliability studies including at least two assessments by the same rater were included. For efficacy studies, only randomized-controlled-trials (RCT) or crossover studies on unhealthy subjects (any condition, duration and outcome) were included. Risk of bias was determined using a modified version of the quality appraisal tool for studies of diagnostic reliability (QAREL) in reliability studies. For the efficacy studies, the Cochrane risk of bias tool was used to assess their methodological design. Two authors performed data extraction and analysis.

**Results:**

Eight reliability studies and six efficacy studies were included. The analysis of reliability studies shows that the diagnostic techniques used in visceral osteopathy are unreliable. Regarding efficacy studies, the least biased study shows no significant difference for the main outcome. The main risks of bias found in the included studies were due to the absence of blinding of the examiners, an unsuitable statistical method or an absence of primary study outcome.

**Conclusions:**

The results of the systematic review lead us to conclude that well-conducted and sound evidence on the reliability and the efficacy of techniques in visceral osteopathy is absent.

**Trial registration:**

The review is registered PROSPERO 12th of December 2016. Registration number is CRD4201605286.

**Electronic supplementary material:**

The online version of this article (10.1186/s12906-018-2098-8) contains supplementary material, which is available to authorized users.

## Background

The practice of osteopathy was founded in 1874 by Andrew Taylor Still in the USA [1]. For the World Health Organization (WHO), osteopathy is a complementary and alternative medicine consisting of manual techniques for diagnosis and treatment for diverse conditions (such as musculoskeletal and gastrointestinal complaints) [2]. Reliable empirical data concerning different types of techniques used in osteopathic practice are rare, essentially due to the poor representativeness of the samples studied. Among all patients treated by osteopaths, the number receiving visceral osteopathy varies widely, from 1% to 95% [3, 4]. Despite the fact that teaching of visceral osteopathy has been banned in some countries (e.g.*,* France [5]), the WHO incorporated visceral techniques in its benchmarks for training in osteopathy in 2010 [2]. However, the introduction of a discipline into clinical benchmarks and more generally into health care systems should require rigorous proofs of safety, efficacy and quality assurance. To fulfill this standard, the patient diagnostic techniques and the therapies themselves must be shown to be both reliable and effective.

From a historical point of view, the concept of visceral osteopathy was introduced by the French osteopath Jacques Weischenck in the 1980s [6]. The subsequent 1983 publication by the French osteopaths Jean-Pierre Barral and Pierre Mercier [7] is relied upon by most osteopaths.

According to the theory proposed by its founders, visceral osteopathy is essentially described in mechanical terms and focuses on the intra-abdominal organs [6, 7]. Starting from the observation that intra-abdominal viscera naturally move (for example due to breathing), it is argued that this mobility could be disturbed in the same way that articular mobility can be disturbed [7]. From a physiopathological point of view, it is claimed that these disturbances can trigger, increase or maintain musculoskeletal (e.g., low back pain) or gastrointestinal complaints (e.g., irritable bowel disorders) [6, 7], among others. Consequently, visceral osteopaths propose that these mobility disturbances can be detected by palpation and treated by manipulation [6, 7]. Currently, none of the theoretical aspects of visceral osteopathy have received serious empirical support apart from the possibility of disturbance of viscera mobility [8]. Moreover, no systematic review has investigated the evidence of intra- and inter-examiner reliability of the diagnostic techniques used in visceral osteopathy.

One literature review has been performed on the efficacy of therapeutic strategies in visceral osteopathy [9]. This review is unfortunately neither systematic (no research, inclusion, and analysis methods) nor specific to visceral osteopathy because it includes studies on abdominal massage. This paper proposes two systematic reviews to identify and critically appraise the scientific literature concerning 1) the reliability of the diagnostic techniques and 2) the clinical efficacy of techniques used in visceral osteopathy.

## Methods

The protocol was registered on PROSPERO (CRD42016052861: http://www.crd.york.ac.uk/PROSPERO/display_record.asp?ID=CRD42016052861) on 12 December 2016, and followed PRISMA (Preferred Reporting Items for Systematic Reviews and Meta-Analyses) recommendations http://prisma-statement.org.

### Data sources and searches

In December 2017, the following literature sources were searched: MEDLINE, OSTMED.DR, the Cochrane Library, Osteopathic Research Web, Google Scholar, Journal of American Osteopathic Association (JAOA), International Journal of Osteopathic Medicine (IJOM) websites, and the catalog of *Académie d’ostéopathie de France*.

(See Additional file 1: Appendix 1, for search terms and equations).

The search was performed until the 21th of December 2017. No restrictions were applied concerning publication date or publication language.

For the sake of completeness, a complementary search was conducted. It consisted of analyzing the list of references of included articles, reading previous systematic reviews, and contacting professional organizations or the authors of unpublished studies for additional studies.

### Study selection

Only inter-rater reliability studies including at least two raters or the intra-rater reliability studies including at least two assessments by the same rater were included. Furthermore, only studies on humans (healthy or unhealthy subjects) were retained. Concerning the interventions evaluated, all studies regarding techniques mentioned in the classical visceral osteopathic literature or claimed by authors to be in the field of visceral osteopathy were retained. The benefit of doubt was given to techniques of whose membership to visceral osteopathy could not be ascertained clearly.

For efficacy studies, only randomized-controlled-trials (RCT) or crossover studies on unhealthy subjects (any condition, duration and outcome) were included. Concerning the interventions evaluated, the same principle as for reliability studies was applied (see above). Other exclusion criteria were: non-comparative trials, non-crossover study, an absence of a clear mention of the use of visceral osteopathy techniques, and studies in which combined treatments were assessed (as in osteopathic manual therapy − OMT) without performing subgroup analysis, and eventually studies for which the full text version is unavailable. No restriction was made concerning the type of illness, the type of outcomes or the type of healthcare services.

Three stages composed the systematic selection process. Firstly, a selection was made based on the title of the article. Second, each abstract was evaluated. At this stage, studies that did not meet the eligibility criteria were excluded. Finally, full-text articles were read for a last application of the eligibility criteria. One author performed the systematic selection process. For the studies gathered through the complementary approach, their abstracts, or if needed, the full-text articles, were also analyzed.

### Data extraction and quality assessment

Two authors performed the data extraction. The data extracted are: the design of the study (with randomization and blinding procedures), sample size and features (e.g., age and/or disease or inclusion criteria), as well as primary and secondary outcomes. Regarding reliability studies, additional information are presented concerning raters (such as number, qualification or expertise) as well as the statistical analysis carried out. As regards efficacy studies, a brief description of the techniques implemented is also presented.

In accordance with PRISMA recommendations http://prisma-statement.org, risk of bias assessments were made independently by two reviewers with standard forms. In case that the risks of bias cannot be fully assessed due to missing information, the corresponding author of the publication (or failing that, the lead author) was contacted to obtain the information necessary to assess the risks of bias. All authors were contacted on the 20th of December 2017. The authors who did not respond were contacted again on the 28th of December 2017.

Reviewers resolved disagreements through discussion and consensus. When no consensus could be reached, a third reviewer made the decision.

### Reliability studies

As regards reliability studies, the risk of bias was assessed in each study using a modified version of the quality appraisal tool for studies of diagnostic reliability (QAREL) [10]. [Note: In comparison to our previous systematic review [10], we withdraw the item “rater’s experience” because an end-study student may be better trained than a recently graduated practitioner. Moreover, it is always possible to carry out a subgroup statistical analysis to assess a potential expertise effect. It should be noted that the withdrawal of this item does not change the conclusion for our previous review on cranial osteopathy [10]]. The general assessment of risk of bias for a reliability study is: ‘High risk of bias’ if at least one item is assessed with a high risk of bias; ‘Major doubt about risk of bias’ if more than two items are assessed with an unclear risk of bias and with all other items with a low risk of bias; ‘Minor doubt about risk of bias’ if two or fewer items are assessed with an unclear risk of bias and with all others with a low risk of bias; and overall ‘Low risk of bias’ if all items are assessed with a low risk of bias [10].

In addition to the general assessment of risk of bias, the results of reliability studies are analyzed and interpreted. The reliability is considered to be satisfactory when the intraclass correlation coefficient (ICC) is above 0.75, according to the Fleiss’ classification, or when the kappa coefficient (κ) is above 0.81, according to the Landis & Koch classification [11, 12]. The targets set could be regarded as high for manual techniques. However, as visceral osteopathy is mainly founded on a causal hypothesis without evidence, these precautions were deemed to be required.

Concerning statistical methods for reliability studies, according to Lucas et al. [13], it is considered that intraclass correlation coefficient, is appropriate for rating inter-rater reliability when the variables are quantitative, ordinal, interval, and ratio variables, while kappa coefficient is appropriate for nominal (i.e., categorical) variables. Other statistical measures of reliability exist, such as Spearman or Pearson correlations, percentage agreement or measures of precision (for example, confidence limits), but they are not adapted for measuring reliability [13, 14].

### Efficacy studies

For efficacy studies, the risk of bias was assessed by means of the Cochrane risk of bias tool [15]. Considering that a high risk of bias in the blinding domain is inevitable in the manual therapy field, the general risk of bias is [10]: ‘High risk of bias’ if at least one item in addition to of “blinding” is assessed with a high risk of bias; ‘Major doubt of risk of bias’ if two or more items are assessed with an unclear risk of bias and with all other domains (aside from blinding) being assessed with a low risk of bias; ‘Minor doubt of risk of bias’ if only one item is assessed with an unclear risk of bias, and with all others (aside from blinding) being assessed with a low risk of bias; and ‘Low risk of bias’ if all items other than blinding are assessed with a low risk of bias.

### Role of the funding source

This systematic review was funded by the French national council of physiotherapists [Conseil national de l’ordre des masseurs-kinésithérapeutes français]. The French national council of physiotherapists had no role in study design; collection analysis, or interpretation of data; or writing of the report.

## Results

### Reliability studies

**Fig. 1:**
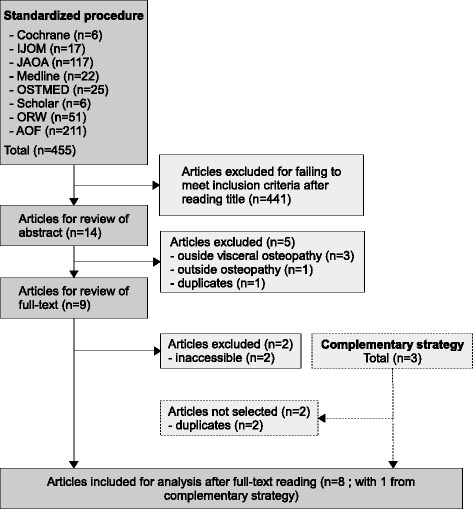
Flow chart of the study selection process for the systematic review of studies dealing with the reliability of diagnosis in the field of visceral osteopathy

455 articles were identified after the standardized bibliographic search. Of these, eight reached the inclusion criteria (Fig 1). The complementary approach gave three additional articles but only one reaching the inclusion criteria. Features of these studies are available in Table 1. Articles excluded after examination of the full text are available in Additional file 2: Appendix 2 with their main reason for exclusion.

**Table 1:** Summary of included studies dealing with the reliability of diagnosis in visceral osteopathy

First authors	Subjects (number; disease status; age in yrs)	Raters (number; degree(s); expertise)	Study Characteristics & Parameter(s)	Reliability Measure Used	Main Results
Landry [16]	*N* = 41; healthy; A = 24.	*N* = 2; last-year osteopathy students; clinical internships.	Inter-rater: (1) proximal-duodenum-area mobility; (2) distal-duodenum-area mobility (4 possible modalities for (1) & (2)).	Cohens’s kappa	Inter: (1): 0.14 (2): 0.0448
Terrier [17]	*N* = 50; patients in osteopathic cares; A = 33	N = 2; last-year osteopathy students; clinical internships.	Inter-rater: ascendant-colon-area dynamic (4 possible modalities).	Cohens’s kappa	Inter: 0.322
Rittler [18]	*N* = 18; osteopathy students with an “osteopathic dysfunction”; not reported.	*N* = 6; osteopaths graduated in 2009; 5 use usually the assessed test and 1 has never used it.	Intra & inter-rater: posture variations (“global listening test”; 6 modalities).	Cohens’s kappa	Intra: from − 0.05 to 0.12 Inter: from − 0.25 to 0.37
Gruber [19]	*N* = 43; 20 healthy and 21 spine-painful patients; A = 30–75	*N* = 2; osteopaths graduated in 2009; 6 yrs.	Intra & inter-rater: abdominal diaphragm tension (2 modalities: symmetrical or asymmetrical).	Cohens’s kappa	intra: from − 0.02 to 0.57 inter: − 0.35
Cònsol Urgellés [20]	*N* = 40; not reported; A = 33	N = 3; osteopaths; recently graduated.	Intra & inter-rater: (1) radial pulse evolution during the Adson-Wright test (or Soto-Hall test) (3 modalities: presence, decrease or abolition); (2) When (1) is “decrease” or “abolition”, location of the “visceral osteopathic dysfunction”.	Fleiss’kappa	Intra: (1) from 0.65 to 1; (2) from 0.63 to 1 Inter: (1) from 0.61 to 0.70; (2) from − 0.14to 0.61
Zeller [21]	*N* = 44; 24 patients with an “asymptomatic hepatic dysfunction” and 20 with a symptomatic hepatic issue^a^; A = 27–73.	N = 2; osteopath-physiotherapists; 2 yrs. in osteopathy and over 10 yrs. in physiotherapy.	Inter-rater: hepatic-area mobility (4 modalities).	Cohens’s kappa	Inter: 0.26
Darty [22]	*N* = 10; healthy; A = 23	*N* = 12; 5 osteopaths and 7 last-year osteopathy students; not reported.	Intra & inter-rater: (1) sensibility; (2) “wall depressibility”; (3) “organ depressibility”; (4) “organ location” ((1),(2), (3), (4) for stomach, caecum, sigmoid and transverse colon; 4 modalities for (1), (2), (3) and distance measure for (4)); (5) “organ volume” (metrical measure; not for stomach).	Intra: variation coefficient (VC) Inter: Student test, correlation coefficient and ICC^a^.	Intra: (1) & (2) not reported; (3) VC < 1% (all the organs); (4) 10 ≤ VC ≤ 125.11 depending on the organ; (5) 21.13 ≤ VC ≤ 38.12 depending on the organ. Inter^b^: (1) & (2) not reported; (3) ICC > 0.9 for all the organs; (4) 0,4 ≤ ICC ≤ 0.98 depending on the organ; (5) 0.64 ≤ ICC ≤ 0.99 depending on the organ.
Verbaarschot [23]	*N* = 31; healthy; A = 17–69	N = 2; osteopaths; specifically trained for the study.	Intra-rater of: visceral tension (3 modalities: normal, “hypertension” or “hypotension”).	Cohens’s kappa	Intra: from 0.372 to 0.542

*Legend*: “*N*” number; “*A*” age; “*ICC*” intraclass correlation coefficient*.* ^*a*^ *No more information are given.*^*b*^ *Only the ICC are reported because the other measures are not recommended for reliability* [16]

**Fig. 2:**
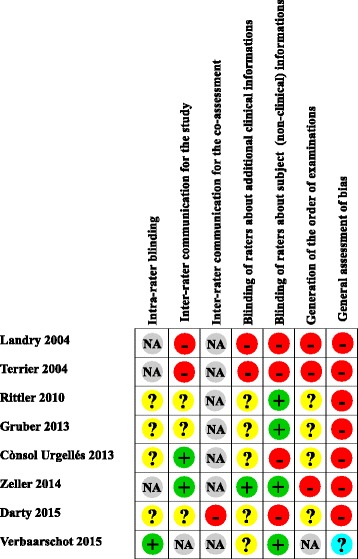
Assessment of methodological risk of bias for each reliability studies included. Green shading indicates a low risk of bias, yellow an unclear risk of bias and red a high risk. Grey shading color indicates non-applicable items. For general assessment of bias, purple shading and cyan shading indicates a major doubt and a minor doubt as to the overall risk of bias, respectively

**Fig. 3:**
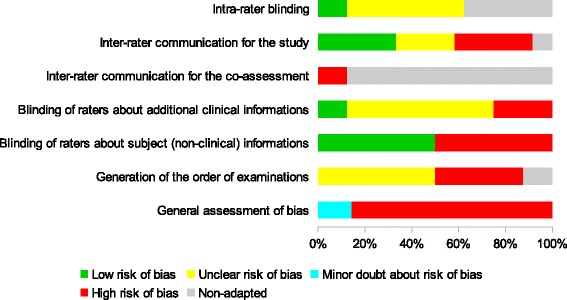
Assessment of methodological risk of bias for the reliability studies taken together. Green shading indicates a low risk of bias, yellow an unclear risk of bias and red a high risk. Grey shading color indicates non-applicable items. For general assessment of bias, purple shading and cyan shading indicates a major doubt and a minor doubt as to the overall risk of bias, respectively

Critical evaluation led to conclude that one study demonstrated a minor doubt about risk of bias [23] and that all other reliability studies had a high risk of bias, especially owing to the absence of blinding of the raters (Figs. 2 & 3). [Note: For the reliability studies, five points are concerned by blinding. The first point is “the prior findings of the test under evaluation” [13]. The second point, which only concerns inter-rater reliability studies, is the possibility of communication between the two raters during the study [13]. The third point is the possibility of communication between the two raters when a subject is tested at the same time by both raters [10]. The fourth point is the “Knowledge of clinical information provides indirect knowledge of the presence or absence of the target disorder or variable of interest and may influence a rater’s decision regarding the outcome of the test.” [13]. For example, clinical history. The fifth point is the “additional cues that are not part of the test” [13], such as tattoos, surgical scars or voice accent.] Seven studies dealt with inter-rater reliability [16–22] and 5 with intra-rater reliability [18–20]. Among the inter-rater reliability studies, 3 addressed visceral mobility and all three showing unreliable results [16, 17, 21] The other studies were designed to evaluate different outcomes such as postture variations [18], abdominal diaphragm tensions [19], the location of a “visceral osteopathic dysfunction” [20] or organ depressibility [22], with three failing to demonstrate reliability [18–20] and one with selective report [22]. The five studies dealing with intra-rater reliability focused on the same outcomes mentioned previously [18–20, 22] plus one study investigating “visceral tensions” [23]. They obtained similar results to the inter-rater reliability studies.

### Efficacy studies

**Fig. 4:**
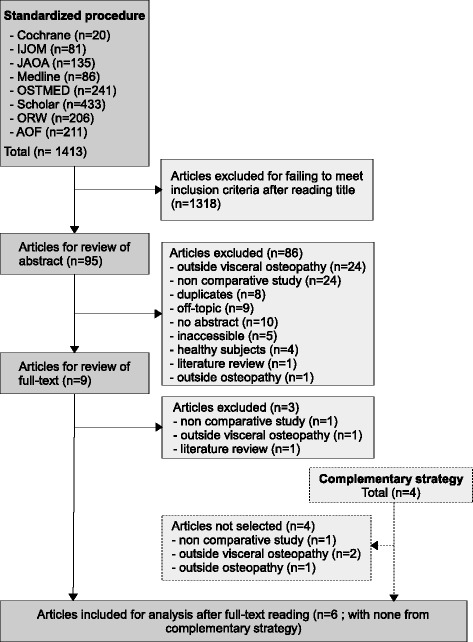
Selection process for studies dealing with the clinical efficacy of techniques and therapeutic strategies used in visceral osteopathy

**Fig. 5:**
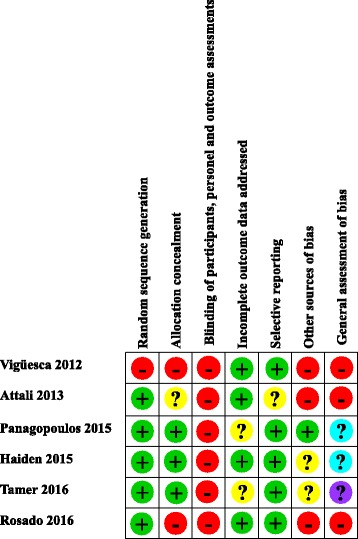
Assessment of methodological risk of bias for each efficacy studies included. Green shading indicates a low risk of bias, yellow an unclear risk of bias and red a high risk. Grey shading color indicates non-applicable items. For general assessment of bias, purple shading and cyan shading indicates a major doubt and a minor doubt as to the overall risk of bias, respectively

**Fig. 6:**
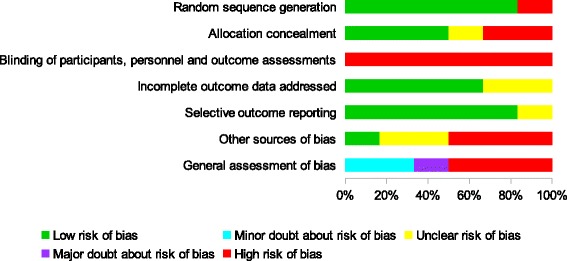
Assessment of methodological risk of bias for the efficacy studies taken together. Green shading indicates a low risk of bias, yellow an unclear risk of bias and red a high risk. Grey shading color indicates non-applicable items. For general assessment of bias, purple shading and cyan shading indicates a major doubt and a minor doubt as to the overall risk of bias, respectively

1413 articles were identified after the standard search procedure. Of these, six reached the inclusion criteria (Fig 4). In the complementary approach 4 additional articles were found with none meeting the inclusion criteria. Features of these six studies are available in Table 2. Articles excluded after examination of the full text are available in Additional file 2: Appendix 2 with their main reason for exclusion. Critical evaluation led to conclude that 3 studies demonstrated a high risk of bias [27–29], one had a major doubt concerning the risk of bias [26] and 2 was assessed with a minor doubt regarding the risk of bias [24, 25] (Figs 5 & 6). Additional issues in studies with high risk of bias are found, such as the lack of a primary outcome [26–29], no Bonferroni corrections were implemented to control for inflated alpha values [25–29], the absence of interpretation of the clinical relevance of the results, no comparison between treatments, and subjective assessment with an unclear blinding procedure [25–29].

**Table 2:** Description of included studies dealing with the clinical efficacy of techniques used in visceral osteopathy

Risk of bias	First author	Disease & number of participants	Intervention and comparison	Primary study outcome & result	Other outcomes & results
Minor doubt about risk of bias	Panagopoulos [24]	Low back pain: 64	EG “standard physiotherapy + visceral manipulation”/ PG “standard physiotherapy + placebo visceral manipulation”	Pain intensity (*0–10 Numerical Pain Rating Scale*) at 6 wks. Results show no SSD.	• 8 criteria (3 outcomes after 2 wks, 2 ones after 6 wks and 3 ones after 1 yr). 1. Pain intensity (0–10Numerical Pain Rating Scale). 2. Disability (Rolland-Morris Disability Scale). 3. Function (Patient-Specific Functional Scale). • Results show no SSD for all criteria with the exception of pain intensity after 1 yr. [PG: 2.17; EG: 3.73].
	Haiden [25]	Very low birth weight infant: 41	EG (visceral osteopathy; “protocol adapted from visceral treatment of adults by Barral and Finet”) / untreated G	The time to complete meconium evacuation. Results show no SSD.	4 criteria 1. Introduction of enteral feeding in days. 2. Feeding volume on day 14th. 3. Time of full enteral feeding in days. 4. Hospital stay in days. • Results show no SSD for all criteria with the exception of time of full enteral feeding, in favor of the untreated G [EG: 34; untreated G: 26]
	Tamer [26]	Nonspecific low back pain: 39	One G treated by OMT + physiotherapy (OMT G) / one G treated by OMT + visceral techniques (vOMT)	None	• 12 criteria (12 outcomes immediately after treatment). 1. Pain intensity (Visual Analogic Scale). 2. Function level (*Oswestry Function Scale*). 3. Physical function (*SF-36 for quality of life*). 4. Physical role limitation (*SF-36 for quality of life*). 5. Pain (*SF-36 for quality of life*). 6. General health (*SF-36*). 7. Energy (*SF-36*) 8. Social function (*SF-36*). 9. Emotional role limitation (*SF-36*). 10. Mental health (*SF-36*). 11. Total physical score (*SF-36*) 12. Total mental score (*SF-36*) • Results show SSD for 3 criteria in favor of the vOMT: physical function [OMT G: 30; vOMT: 46]; energy [OMT G: 10; vOMT: 22]; total physical score [OMT G: 10.5; vOMT: 20.6].
High risk of bias	Vigüesca [27]	Sacroiliac pain: 14	EG “ileocecal valve inhibition technique”/ untreated G	None	• 6 criteria (2 outcomes immediately after treatments and 1 wk. and 2 wks later). 1. Pain intensity (*Visual Analogic Scale*). 2. Functional disabiltity (*Oswestry Disability Index*). • Results show difference between pretest and post-test for pain intensity [untreated G: 0; EG: 0.72] and functional disability [untreated G: 0.15; EG: 5.0], and 2 wks later again for pain intensity [untreated G: 0.15; EG: 1.54]. Selective reporting of results.
	Attali [28]	Irritable bowel syndrome (IBS): 31	Cross-over range of 10 wks with 2 G (standard therapy + visceral osteopathic manipulation vs. Standard therapy + “placebo manipulation”)	None	45 criteria (21 outcomes 3 wks and 6 wks after treatments, and 3 outcomes 1 yr. later). 1. Constipation (*Visual Analogic Scale*). 2. Diarrhea (*Visual Analogic Scale*). 3. Abdominal distension (*Visual Analogic Scale*). 4. Abdominal pain (*Visual Analogic Scale*). 5. Colonic transit time: in the right & left colon and the recto-sigmoid (*monitoring of ingested radiopaque markers*). 6. Rectal sentivity: threshold sensation volume, constant sensation volume and maximum tolerable volume (*Anorectal manometry*). 7. Pain intensity in 9 abdominal areas (*Visual Analogic Scale*). 8. Presence or absence of depression (*two-question case-finding instrument*). 9. Evolution of IBS phenotype (“*Standardized questionnaire as defined by the Rome III criteria*”). • Results: selective reporting of results. Number of criteria above 15^a^.
					• 1. 2. 3. 4. 5. 6. 7. 8. 9. •
					• 1. 2. 3. 4. 5. •
	Rosado [29]	Irritable bowel syndrome: 40	EG (Osteopathic visceral manipulation) / “placebo treatment”	None	12 criteria (6 outcomes immediately after treatments and 1 wk. later). 1. Lumbar range of motion (LROM) in flexion (Goniometry) 2. LROM in extension (Goniometry) 3. LROM in right side bending (Goniometry) 4. LROM in left side bending (Goniometry) 5. LROM in flexion (*Modified-Modified Schober Test* − MMST) 6. LROM in extension (MMST) • Results: no inter-group statistical comparison; outcomes improve for the two groups apart from the LROM in extension measured by the MMST.

*Legend. EG experimental group, G group, SSD significant statistic difference, PG placebo group, OMT osteopathic manual therapy, LROM Lumbar range of motion, MMST Modified-Modified Schober Test*

^a^ *Considering the risk of inflated alpha value and for sake of clarity, the results of the studies that both had not chosen primary study outcomes and had used more than 15 criteria were not reported*

## Discussion

The aim of this review has been to identify and critically appraise the scientific studies regarding the reliability of the diagnostic techniques and the clinical efficacy of therapeutic techniques used in visceral osteopathy.

No evidence is found for the reliability of diagnostic techniques used in visceral osteopathy. Most studies present a high risk of bias and fail to show reliability for evaluated outcomes. Given that the different biases detected (especially the absence of examiner blinding and absence of randomized examination order) should lead to an artificially increased measured reliability [13], this strengthens the argument that the diagnostic techniques are really unreliable.

As regards the efficacy of visceral techniques, only studies with a low risk of bias or with minor doubts concerning the risk of bias are discussed and considered as evidence. In total, two studies are discussed below.

First, the study of Panagopoulos et al. [24], is a randomized blind controlled trial designed to evaluate the efficacy of visceral manipulation in addition to “standard physiotherapy”, compared with “standard physiotherapy alone”, for low back pain. The main outcome is correctly defined and clinically significant, even though self-reported pain is a subjective criterion. The results demonstrate no significant statistical difference for the main outcome (pain at 6 weeks). Among the eight secondary outcomes, a clinically significative difference is found in favor of the experimental group in only one (pain at 52 weeks). This result could present a motivation for a new study with pain at 52 weeks as main outcome.

Second, the study carried out by Haiden et al. [25], is designed without a placebo treatment to compare the effects of visceral osteopathy in addition to standard care with very low birth weight infants. One primary outcome (time to complete meconium evacuation in days) and four secondary outcomes are proposed. Visceral osteopathy is shown not to be more effective than the case of no additional treatment in the acceleration of meconium passage and the enhancement of feeding tolerance in very low birth weight infants. Moreover, in the experimental group, durations of hospital stay and full enteral feeding were respectively 34 and 10 days longer than in the untreated group. Although it is not possible to deduce adverse effects of visceral osteopathy from this study alone, the absence of placebo treatment in the control group allows its interpretation as unfavorable evidence for the efficacy of visceral osteopathy in this specific context.

In summary, the two studies analyzed do not support the efficacy of visceral techniques in low back pain and for very low birth weight infants.

As a whole, the systematic review presented above shows that most studies addressing the reliability or the efficacy of visceral osteopathy have a high or unclear risk of bias. Therefore, there is insufficient data available to significantly inform the practice of manual therapists. These results are consistent with the last review on cranial osteopathy [10] and several reviews on osteopathic manipulative treatment (OMT) [30–33]. They highlight the requirement to enhance the standards of research methodology in osteopathy. Consequently, as undertaken for clinical research on cranial osteopathy [10], some guidance is provided to yield unbiased methodological studies and to improve the quality of study reporting in visceral osteopathy:

first, given that all reliability and two out of six efficacy studies analyzed conducted by osteopathy students, and that all these studies are rated with a high risk of bias or a major doubt about risk of bias, it is recommended to avoid (for now) studies conducted by osteopathy students in future systematic reviews, and we note room for improvement in both education and supervision of osteopathy students.

Second, in studies included in the review, most items are assessed with unclear risk of bias. This state of affairs could be improved if more methodological details were given by authors. However, it can be argued that the length of the articles is limited in many academic journals. Unfortunately, authors often opt for shortening the method section, reducing the possibility to detect potential bias. Therefore, it can be recommended either to publish separate methodology papers or to add such details as appendices or supplementary material.

Third, regarding the reliability studies, it can be recommended to future researchers in visceral osteopathy to draw inspiration from the items proposed in this systematic review and based on the QAREL. For inter-rater reliability studies, close attention must be paid to avoid raters sharing information during the entire duration of the study, consequently, studies spanning over more than 1 day are not recommended. The possibility of information-sharing between examiners requires procedures to avoid memorization of examination results. First, only the minimum requirement of clinical information concerning subjects should be given to raters and the assessment sequences (subjects and raters) should be randomized. Moreover, blinding of examiners and subjects has to be as rigorous as possible. Halma et al. [34], in the field of cranial osteopathy, for instance, implemented a suitable method to isolate the rater from visual, auditory, tactile, and olfactory cues. It should also be noted that for studies implying simultaneous assessments by two raters, the methods carried out by Rogers et al., [35], Moran & Gibson [36], and Sommerfeld et al. [37], in cranial-osteopathy studies, could be used as a guidance for this methodological aspect.

Finally, future researchers are advised to use the Cochrane risk of bias tool to design a well-built efficacy study. Moreover, the 2010 CONSORT checklist can help to implement a rigorous randomized controlled clinical trial. The exemplary methodological precautions adopted by Panagopoulos et al. [24], but also, in the field of cranial osteopathy, by Elden et al. [38], and Haller et al. [39], deserve to be underlined. However, all three studies have a bias not only because the therapeutic procedures differ (in duration, practitioner, etc.) between groups. Hence, this bias creates a risk of confusion between specific and contextual effects. To avoid this bias, future researchers should rigorously standardize the different therapeutic procedures regarding the number and duration of sessions, practitioner-patient relationship, etc. Furthermore, most studies did not assess the credibility of the placebo used. Such an assessment should be conducted in future studies in order to compensate for the insufficient blinding procedure specific to the field. Finally, when designing a study it is important to specify one main outcome, rather than making multiple comparisons. If several outcomes are chosen, a statistical correction should be planned to offset the alpha risk inflation.

## Conclusion

As a whole, this systematic review shows that currently, there is no evidence for the reliability or specific efficacy of the techniques used in visceral osteopathy. These results are consistent with the last review on cranial osteopathy and highlight the requirement to enhance research methodological standards in manual therapies, particularly in osteopathy.

## Additional files

Additional file 1: Detailed search strategy for reliability and efficacy studies. Description of data: Appendix 1 contains literature sources, search terms and equations of the systematic review. (ODT 22 kb)

Additional file 2: Excluded articles after full-text examination. Description of data: Appendix 2 contains articles excluded after examination of the full text with their main reason for exclusion. (ODT 18 kb)
