# Supplementary material for: Reliability of diagnosis and clinical efficacy of visceral osteopathy: a systematic review
Source: BMC Complement Altern Med. 2018 Feb 17;18:65. doi: 10.1186/s12906-018-2098-8 (PMC5816506; doi:10.1186/s12906-018-2098-8)
Supplement: Supplementary file 1 [file 12906_2018_2098_MOESM1_ESM.odt]

Detailed search strategy for reliability and efficacy studies

Detailed search strategy for reliability studies

- Pubmed

The following formula was used in the classic search bar:

(reliability [ti] OR agreement [ti] OR reproducibility [ti]) AND (osteopathy [tiab] OR osteopathic [tiab]) 

- OSTMED.DR

Advanced search→ two search lines added by the option Add another field → use of the keywords: reliability, agreement et reproducibility → search field Title → keywords associated by the boolean operator OR.

- Cochrane Library

Advanced search → formula: ("reliability" OR "reproducibility" OR "agreement") AND (osteopathy OR osteopathic) - default search field: Title, Abstract, Keywords.

- IJOM

Advanced search → one search lines added by the option Add another search term→ use of the keywords reliability, reproducitbility et agreement → search field Title → keywords associated by the boolean operator OR.

- JAOA

Advanced search → formula: reliability reproducibility agreement

(Option) Search for: Any

- Google Scholar

Classic search bar → formula: allintitle:((reliability OR reproducibility OR agreement) AND (osteopathy OR osteopathic)) → after the search was done, disabling of patents and quotations.

- Osteopathic Research Web

Advanced search → keywords reliability, reproducibility, agreement in three different search fields (without the commas) → keywords associated by the boolean operator OR → search field Title word for each keyword; option Show only items with abstracts.

- Académie d'ostéopathie de France

(Former interface of the library catalog)

- Basic search → keyword viscérale in the search field → option Tous les champs

- Basic search → keyword viscéral in the search field → option Tous les champs

Detailed search strategy for efficacy studies

- Pubmed

The following formula was used in the classic search bar:

"visceral osteopathy" OR "visceral manipulation" OR "visceral field" OR "organ manipulation" OR "abdominal technique"

- OSTMED.DR

Five searches were carried out:

- Advanced search→ keyword: visceral → (in) field Title.

- Advanced search→ keyword: visceral → (in) field Description.

- Advanced search→ two search lines added by the option Add another field → keywords: ventral, abdominal, organ → search field Description for each keyword → keywords associated by the boolean operator OR.

- Advanced search→ two search lines added by the option Add another field → keywords: ventral, abdominal, organ → search field Title for each keyword→ keywords associated by the boolean operator OR.

- Cochrane Library

Advanced search → formula: "visceral osteopathy" OR "visceral osteopathic" OR "visceral manipulative" OR "visceral manipulation" OR "visceral manual" OR "abdominal technique" - default search field: Title, Abstract, Keywords.

- IJOM

Two searches were carried out:

- Classic search bar→ keyword: visceral

- Advanced search→ three search lines added by the option Add another search term → keywords: ventral, abdominal, organ (one keyword per line)→ search field Article title, abstract, keywords  for each keyword → keywords associated by the boolean operator OR.

- JAOA

Two searches were carried out:

- Classic search bar → keyword: visceral 

-  Advanced search → formula: ventral abdominal organ

- (Option) Search for: Any

- Google Scholar

Two searches were carried out:

- Classic search bar → formula: allintitle:"visceral osteopathy" OR "visceral osteopathic" OR "visceral manipulation" OR "organ manipulation" OR "visceral field" OR "visceral technique" OR "visceral manual" OR "visceral treatment" OR "ventral technique" OR "abdominal technique"→ after the search was done, disabling of patents and quotations.

- Classic search bar → formula: "visceral osteopathy" OR "visceral osteopathic" OR (("visceral manipulation" OR "organ manipulation" OR "visceral field" OR "visceral technique" OR "visceral manual" OR "visceral treatment" OR "ventral technique" OR "abdominal technique") AND osteopathy)→ after the search was done, disabling of patents and quotations

- Osteopathic Research Web

Two search were carried out:

- Classic search bar → keyword: visceral

- Advanced search → keywords ventral, abdominal, organ in four different search fields (without the commas) → keywords associated by the boolean operator OR → search field Abstract word for each keyword; option Show only items with abstracts.

- Académie d'ostéopathie de France

(Former interface of the library catalog)

- Basic search → keyword viscérale in the search field → option Tous les champs

- Basic search → keyword viscéral in the search field → option Tous les champs
