# Supplementary material for: Reliability of diagnosis and clinical efficacy of visceral osteopathy: a systematic review
Source: BMC Complement Altern Med. 2018 Feb 17;18:65. doi: 10.1186/s12906-018-2098-8 (PMC5816506; doi:10.1186/s12906-018-2098-8)
Supplement: Supplementary file 2 [file 12906_2018_2098_MOESM2_ESM.odt]

Excluded articles after full-text examination

Excluded articles after full-text exmamination for reliability studies

| First authors (year) | Title | Main exclusion motive |
| --- | --- | --- |
| Beal & Patriquin (1995) | Interexaminer agreement on palpatory diagnosis and patient self-assessment of disability: a pilot study | Outside visceral osteopathy |
| Robyr (2004) | Étude pilote sur la concordance de tests des zones de reins dans leurs paramètres d’inspiration et d’exspiration par deux étudiantes stagiaires sur des patients en consultation en cabinets privés. (French) | Inacessible[^1] |
| Gau et al. (2009) | Interexpert agreement on diagnosis of bacteriuria and urinary tract infection in hospitalized older adults: My First Collection | Outside osteopathy |
| Degenhardt et al. (2010) | Maintenance and improvement of interobserver reliability of osteopathic palpatory tests over a 4-month period | Outside visceral osteopathy |
| Körner (2015) | Testing the interrater reliability of osteopathic findings in the examination of the abdominal diaphragm without specification of test implementation | Inaccessible[^2] |

Excluded articles after full-text exmamination for efficacy studies

| First authors (year) | Title | Main exclusion motive |
| --- | --- | --- |
| Horton (2015) | The anatomy, biological plausibility and efficacy of visceral mobilization in the treatment of pelvic floor dysfunction | Literature review |
| Orofino et al. (2015) | Osteopathy and improving the quality of life in crohn 's disease | Outside visceral osteopathy |
| Bramati-Castellarin et al. (2016) | Repeat-measures longitudinal study evaluating behavioural and gastrointestinal symptoms in children with autism before, during and after visceral osteopathic technique (VOT) | Non comparative study |

[^1]: We received no response from the author after an e-mail and a reminder.
[^2]: The same as the previous footnote.
